# Randomized clinical study on radial artery compression time after elective coronary angiography*

**DOI:** 10.1590/1518-8345.2584.3084

**Published:** 2018-11-29

**Authors:** Maria Aparecida de Carvalho Campos, Claudia Maria Rodrigues Alves, Miriam Harumi Tsunemi, Maria Angélica Sorgini Peterlini, Ariane Ferreira Machado Avelar

**Affiliations:** 1 Universidade Federal de São Paulo, Hospital São Paulo, São Paulo, SP, Brasil.; 2 Universidade Estadual Paulista Júlio de Mesquita Filho, Instituto de Biociências, Botucatu, SP, Brasil.; 3 Universidade Federal de São Paulo, Escola Paulista de Enfermagem, São Paulo, SP, Brasil.

**Keywords:** Cardiac Catheterization, Occlusion, Radial Artery, Hemostasis, Complications, Nursing

## Abstract

**Objective:**

to compare two compression times of the radial artery after coronary angiography with customized compressive dressing regarding the occurrence of hemostasis and vascular complications.

**Method:**

a randomized clinical study was carried out in patients undergoing elective transradial coronary angiography in two study groups: (G30), whose compressive dressing was maintained for 30 minutes, and (G60), whose compressive dressing was maintained for 60 minutes, both until the first evaluation of hemostasis. Variables related to patients, procedure, occurrence of hemostasis, and vascular complications were analyzed. Patency of the radial artery was assessed with Doppler vascular ultrasonography, immediately after removing the compressive dressing and 30 days after the procedure.

**Results:**

the sample consisted of 152 patients in G30 and 151 in G60. Hemostasis was evidenced in the first evaluation in 76.3% of G30 patients and 84.2% of G60 patients (p = 0.063). There were 91 immediate complications, being 53 hematomas and 38 occlusions of the radial artery. We identified 18 late occlusions, 7 (5.5%) in G30 and 11 (8.2%) in G60.

**Conclusion:**

the different compression times of the radial artery after coronary angiography did not significantly influence the occurrence of hemostasis and vascular complications. Brazilian Registry of Clinical Trials (Rebec): RBR-7VJYMJ.

## Introduction

The technique of transradial approach (TRA) gained importance as a strategy for the reduction of vascular complications and severe bleeding episodes in patients undergoing invasive diagnostic and therapeutic coronary procedures, with a potential impact on the reduction of mortality, especially in patients with acute coronary syndrome (ACS) when compared to the femoral artery access technique^(^ ^1^ ^-^ ^5^ ^)^.

It has proved to be an alternative to the traditional femoral artery access to promote greater comfort to patients after the procedure, possibility earlier ambulation, shorter hospital stay time and lower costs^(^ ^6^ ^-^ ^7^ ^)^. Among the complications related to TRA, radial artery occlusion (RAO) is the most common. This complication is poorly diagnosed, generally asymptomatic and with an estimated incidence of one to 10% of patients. Its occurrence makes it impossible to use the radial artery as an access option in further procedures such as a free graft for patients undergoing myocardial revascularization. Factors such as incompatibility between the diameter of the introducer sheath and diameter of the radial artery (RA), insufficient anticoagulation, and interruption of radial artery flow during and after the procedure are considered predictors of RAO. On the other hand, the patent hemostasis after the procedure, i.e. correct pressure during compression, balancing hemostasis and maintenance of anterograde flow; the reduction in compression time; and the use of hemostatic devices to compression of the puncture site of the radial artery are preventive factors for RAO^(^ ^8^ ^-^ ^9^ ^)^.

To the best of our knowledge, few publications in the literature have addressed the influence of the use of compression with customized dressing to obtain hemostasis and the occurrence of vascular complications^(^ ^10^ ^-^ ^11^ ^)^. Most studies compare different dedicated mechanical devices developed by the industry that consist of pneumatic or rotational compression systems with the shape of wristbands^(^ ^12^ ^-^ ^14^ ^)^.

Because of the value added to the cost associated with the use of these dedicated devices, the use of customized compression dressings with gauze cushions for hemostasis at the radial access site is common in developing countries. Without standardization or recommendation based on application evidence, the technique and time of radial artery compression are determined by institutional protocols, backed by clinical experience, but not based on scientific evidence.

Thus, this study aims to compare two radial artery compression times after elective coronary angiography with customized compressive dressing (CCD) regarding the occurrence of hemostasis and vascular complications.

## Method

A prospective, randomized, controlled clinical study was developed at the interventional cardiology unit of a public university hospital offering high complexity care located in the city of São Paulo. The research was conducted from August 2015 to September 2016 after approval of the merit of the research (CAAE46327315.2.0000.5505), acceptance and voluntary agreement of the patients to participate in the study expressed through the signature of the Informed Consent Term. The study protocol was recorded in the Brazilian Registry of Clinical Trials (Rebec): RBR-7VJYMJ and followed the recommendations of the CONSORT group.

Individuals aged 18 years or more who had undergone elective transradial catheterization were included in the study. In turn, the following cases were excluded: patients hemodynamically unstable, presenting a D-type curve in the Barbeau test^(^ ^15^ ^)^; patients who had undergone a previous procedure by ipsilateral radial puncture in case of impossibility to perform coronary angiography due to failure of puncture or non-progression of the catheter; patients who presented vascular complications at the puncture site before the beginning of the hemostasis technique; and patients submitted to percutaneous coronary intervention (PCI) immediately after elective cinecoronariography (ECAT). Patients transferred to the hospitalization unit immediately after the procedure and unable to attend a reassessment after 30 days were also excluded from the study and were categorized as others.

The variables selected for the study included the experimental variable, the control of the experimental variable, variables for characterization of the study groups, and primary and secondary outcome variables.

The experimental variable was considered by the maintenance of CCD, performed with sterile gauze and hypoallergenic adhesive tape, on the radial artery puncture site for 30 minutes until the first evaluation of the occurrence of hemostasis (G30). In the case of bleeding at the first evaluation, further tension was applied in the dressing and additional 30-minute intervals of compression were performed, reevaluating the site every 30 minutes until complete hemostasis.

The control of the experimental variable comprised the dwell of the CCD for 60 minutes until the first evaluation of the puncture site (G60). The choice for 60 minutes in patients of the control group was based on the practice developed at the study site^(^ ^9^ ^)^. In case of bleeding, further tension was applied in the dressing and additional 30-minute intervals of compression were performed, reevaluated the site every 30 minutes until complete hemostasis.

The primary outcome variables were hemostasis (when no evidence of bleeding requiring further compression was detected at removal of the compressive dressing, according to the time established for the study group) and occurrence of vascular complications (local hematoma, pseudoaneurysm, bleeding, compartment syndrome, and immediate and late radial artery occlusion).

Late postoperative complications after removal of the compressive dressing, defined as immediate and late complications, were assessed by means of inspection, palpation and Doppler ultrasonography (USG) with iLook 25^®^ device (SonoSite Inc. , Bothell, USA) for the identification of RAO and the occurrence of pseudoaneurysm. The evaluation of hematoma was based on the classification of the EASY (Early Discharge after Transradial Stenting of Coronary Arteries) study^(^ ^16^ ^)^: type I ≤ 5 cm in diameter; type II ≤ 10 cm; type III > 10 cm, without reaching the elbow; type IV - hematoma extending beyond the elbow; and type V - any hematoma with ischemic injury to the hand.

Secondary outcome variables were analyzed in order to verify the influence of other variables. They were the occurrence of patent hemostasis, defined as hemostasis with maintenance of anterograde flow, as well as additional time for radial artery hemostasis.

All patients with indications for elective transradial coronary angiography were submitted to evaluation of patency of the ulnopalmar circulation before the procedure by the Barbeau test^(^ ^15^ ^)^, in which a pulse oximeter sensor coneected with an Infinity^®^ monitor (Draeger Medical-Pennsylvania-USA) was positioned on the ipsilateral thumb to the limb chosen for examination; the morphology of the plethysmographic waveform was evaluated by the researcher before and two minutes after radial artery compression. The plethysmographic patterns were classified as type A (no curve damping after radial artery compression), type B (curve damping), type C (momentary loss of waveform with recovery up to two minutes) and type D (loss of waveform without recovery of the curve).

In the examination room, the patients were prepared according to institutional protocol. The technique of puncture and the choice of materials used were defined by the professional performing the procedure. After placement of the catheter at the root of the aortic artery, all patients received 50U/kg of unfractionated heparin administered intravenously; the use of spasmolytic drugs was a decision of the medical professional.

After completion of the procedure, patients were randomly allocated to one of the G30 or G60 study groups.

**Figure 1:**
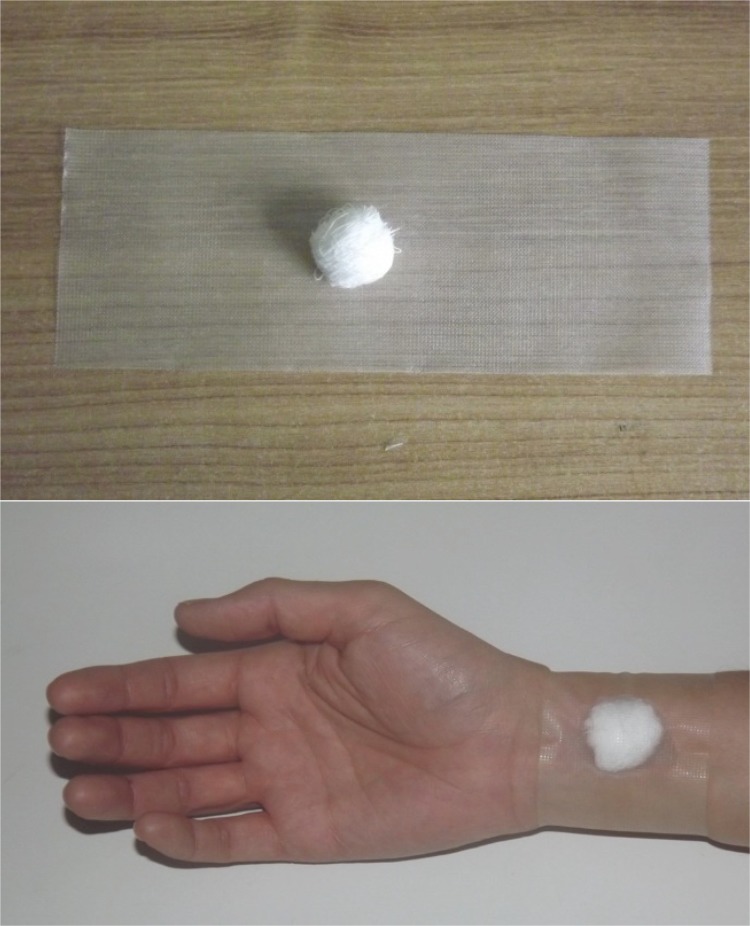
Dressing made with sterile gauze cushion and hypoallergenic adhesive tape, positioned on the radial artery puncture site after removal of the introducer sheath

After inclusion in one of the study groups, the radial introducer was removed by the professional performing the procedure; the compressive dressing prepared with a sterile gauze cushion and hypoallergenic adhesive tape was positioned by the researcher at the puncture site, as specified in Figure 1.

All time intervals were controlled with chronometers and timers.

The evaluation of patent hemostasis was performed immediately after the placement of the compressive dressing. The pulse oximeter connected with an Infinity^®^ monitor (Draeger Medical-Pennsylvania-USA) was positioned on the ipsilateral thumb for evaluation of plethysmographic waveform during compression of the ulnar artery for 30 seconds. The maintenance of the plethysmographic signal indicated the presence of flow in the radial artery, confirming patent hemostasis. Loss of plethysmographic signal indicated no flow in the radial artery. Even in case of no patent hemostasis, there was no manipulation of the compressive dressing.

At the end of the compression time, depending on the study group, the compressive dressing was removed and the clinical evaluation and Doppler ultrasonographic evaluation were performed in the region above and below the puncture site to evaluate the occurrence of vascular complications. Immediate RAO was indicated by the absence of sing of flow in the USG. Patients were discharged with an occlusive dressing at the puncture site and were advised about post-procedure care and also that they should return to care service after 30 days for another clinical evaluation, with inspection and palpation, and ultrasonography of the puncture site, in order to identify the presence of hematoma and RAO.

The following variables of the patients were assessed: sex, age, body mass index (BMI), risk factors (hypertension, dyslipidemia, diabetes mellitus, smoking, family history of coronary disease), current clinical presentation (stable angina, acute coronary syndrome, preoperative evaluation, positive functional evaluation test) and use of medications. ; Variables related to the procedure were also determined, namely, the caliber of the introducer sheath, puncture time, i.e. time elapsed from the moment of local anesthesia to insertion of the introducer sheath, number of puncture attempts, dose of heparin administered, duration of the examination (from the moment of local anesthesia to the removal of the last catheter), occurrence of radial artery spasm, according to patient’s report of pain and resistance perceived by the operator during manipulation of the catheter.

Primary and secondary outcomes were evaluated by the researcher after training in the acquisition and interpretation of ultrasonographic imaging.

The sample size was defined after the post-test with 62 patients, and it was calculated in 246 patients using the Chi-Square test, with a significance level of 0.05 and 95% power, based on the association between each characterization variable of patients and the occurrence of complications.

The participants were randomly allocated according to the randomization list generated by the GraphPad^®^ software, developed by GraphPad Software Inc, San Diego, California, USA.

The variables were entered into a database in a Microsoft Office Excel spreadsheet and statistical analyses were made using the SPSS Statistics version 17.0 (SPSS Inc, Chicago, USA). Continuous variables were expressed using mean and standard deviation or median and interquartile range. Groups were compared using the Student’s t-test for independent samples or the Mann-Whitney test for cases in which the assumption of normality of distribution was not met. Categorical variables were expressed in absolute and relative values and compared using the Pearson’s chi-square or Fischer’s exact test. A significance level of 5% was adopted in all comparisons.

## Results

**Figure 2:**
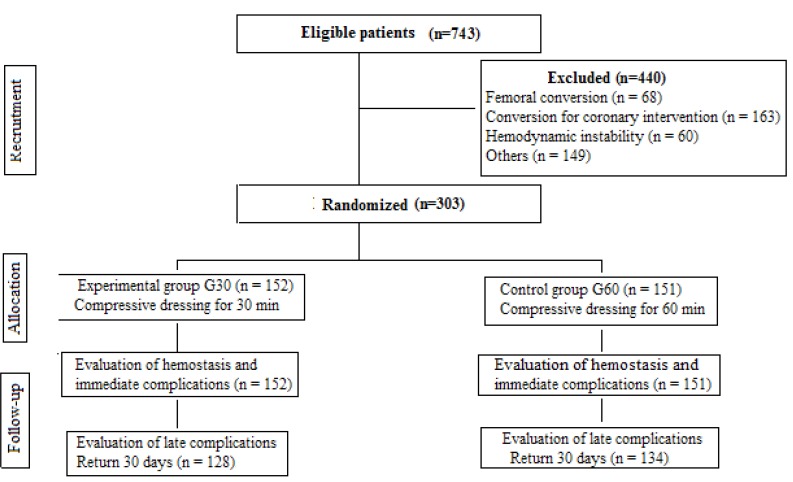
– Diagram according to the *Consolidated Standards of Reporting Trials* (CONSORT)

In the period from August 2015 to September 2016, 743 patients underwent cardiac catheterization diagnosed through the TRA. Of these, 303 patients were included in the study, of which 152 received compressive dressing for 30 minutes (G30) and 151 for 60 minutes (G60). The flowchart of the study is presented in Figure 2.

The sample was characterized by the predominance of males, with a mean age above 60 years, with a diagnosis of stable angina, history of systemic arterial hypertension, and using antiplatelet drugs, beta blockers. The participants had a homogeneous distribution in both groups, except for the body mass index (BMI); this index was significantly higher in the patients of G30 (Table 1).

**Table 1:** – Demographic and clinical characteristics in the experimental (G30)* and control (G60)† groups. São Paulo, SP, Brazil, 2016.

Variables	G30* (n=152)	G60† (n=151)	p-value
	
	n (%)	n(%)	
*(Age years)*			
Median (Q1 - Q3)	62 (54.25 - 68.0)	60 (53.0 - 67.0)	0.4‡
Minimum maximum	27 - 81	25 - 83	
*Sex*			
Male	85 (55.9)	96 (63.6)	0.2§
Body mass index Kg/m^2^	27.7±5.2	26.4±4.2	0.02‡
*Risk factors*			
Arterial hypertension	118 (77.6)	116 (76.8)	0.9§
Dyslipidemia	61 (40.1)	59 (39.1)	0.9§
Diabetes	45 (29.6)	48 (31.8)	0.7§
Smoking	56 (36.8)	58 (38.4)	0.8§
Family history for CAD^││^	07 (4.6)	08 (5.3)	0.8§
No history	10 (6.6)	09 (5.7)
*Current Clinical Presentation*			
Stable Angina	57 (37.5)	55 (36.4)	0.9§
Acute Coronary Syndrome	40 (26.3)	41 (27.1)	0.9§
Preoperative Evaluation	47 (30.9)	43 (28.5)	0.6§
Positive Functional Testing	42 (27.6)	33 (21.9)	0.3§
*Use of medications*			
ASA^¶^	113 (74.3)	114 (75.5)	0.8§
Clopidogrel	52 (34.2)	49 (32.5)	0.7§
Beta blocker	97 (63.8)	103 (68.2)	0.4§
Statins	89 (58.6)	88 (58.3)	0.9§
ACE^**^/CCB^††^	76 (50.0)	83 (55.0)	0.4§

Legend: *G30 - 30 minutes group; ^†^G60 - 60 minutes group; ^‡^Student’s t test for independent samples; ^§^Chi-square test; ^││^CAD - Coronary artery disease; ^¶^ASA - Acetylsalicylic acid; ^**^ACE - Angiotensin-converting-enzyme inhibitor; ^††^CCB - Calcium channel blocker.

There was no significant statistical difference between groups regarding puncture time, number of puncture attempts, procedure time, heparin dose used, occurrence of spasm and number of catheters used per patient. The majority of the patients presented Type B test in the evaluation of the permeability of the palmar arch and they used of 5 French (F) introducer sheath. However, a smaller part of the patients, regardless of the study group, presented patent hemostasis after the positioning of the compressive dressing (Table 2).

**Table 2:** Characterization variables of the procedure in the experimental (G30)* and control (G60)† groups. São Paulo, SP, Brazil, 2016.

	G30^*^(n=152)	G60^†^(n=151)	p-value^‡^
*Introducer sheath caliber, n (%)*			
5 French	93 (61.2)	97 (64.2)	0.6
6 French	59 (38.8)	54 (35.8)	0.6
*Barbeau test, n (%)*			0.6
Type A	33 (21.7)	27 (17.9)	
Type B	116 (76.3)	122 (80.8)	
Type C	3 (2.0)	2 (1.3)	
*Occurrence of spasm, n (%)*	24 (15.8)	20 (13.2)	0.6
Catheter number per patient^§^	1.87±0.9	1.91±0.8	0.4
Number of puncture attempts^§^	1.77±1.31	1.63±1.12	0.3
Puncture time (minutes)^§^	4.32±4.2	4.01±3.27	0.8
Examination time (minutes)^§^	22.9±9.9	23.5±9.3	0.5
Heparin dose (Units)^§^	3711±687	3638±692	0.2

Legend: * G30 - 30 minutes group; † G60 - 60 minutes group; ‡ Chi-square test; ^§^Mean ± standard deviation.

Table 3 presents the results for the primary and secondary outcome variables.

**Table 3:** Occurrence of hemostasis, immediate vascular complication, and complications after 30 days in the experimental (G30)* and control (G60)† groups. São Paulo, SP, Brazil, 2016.

	G30*(n=152)	G60† (n=151)	p-value^‡^
	
	n (%)	n (%)	
*Occurrence of hemostasis*			0.06
Yes	116 (76.3)	128 (84.2)	
No	36(23.6)	23(15.6)	
*Immediate Complications*			
Hematoma			
Type I	15 (9.9)	24 (15.9)	0.1
Type II	7 (4.6)	7 (4.6)	0.9
Immediate RAO^§^	20 (13.2)	18 (11.9)	0.7
Patent hemostasis, n (%)	12 (7.9)	16 (10.6)	0,4

	G30^*^(n=128)	**G60^†^ (n=134)**	**p-valor^‡^**
	
	**n (%)**	**n (%)**	

*Late Complications*			
Hematoma	-	-	
Late RAO§	7(5.5)	11(8.2)	0.4
Average time of RA^││^ compression in patients with late RAO§	40.57±15.10	61.27±2.20	0.01

* G30 - 30 minutes group; † G60 - 60 minutes group; ‡ Chi-square test; ^‡^RAO - Radial artery occlusion, ^││^RA - Radial artery

The occurrence of hemostasis after the stipulated time for the first evaluation according to the study group was evidenced in the majority of the patients. In the other patients that did not reach hemostasis at the time determined for the groups, 36 (23.6%) of the G30 and 23 (15.6%) of the G60, hemostasis was achieved on average at 68.31 (± 12.19) minutes and 96.91 (± 9.33) minutes, respectively.

A total of 91 immediate complications were identified, most of them hematomas that did not require intervention for regression, with a homogeneous presentation in the groups. In the assessment of the radial artery patency immediately after removal of the compressive dressing, RAO was evident in 13.2% of G30 patients and 11.9% of G60 patients (p = 0.75).

In the evaluation performed in the 262 (86.5%) patients who returned after 30 days of the procedure, no hematomas were identified, and there was a reduction in occlusion rates, with no statistically significant difference between the groups.

No other types of vascular complications were recorded on the immediate evaluation and after 30 days.

## Discussion

This study demonstrated that compressive dressing of shorter duration maintained at the puncture site for 30 minutes after elective transradial coronary angiography is as effective and safe as the compressive dressing maintained for 60 minutes with respect to hemostasis and occurrence of immediate and late complications. Hemostasis was evidenced in most patients, according to the times determined for the first evaluation, regardless of the study group.

The occurrence of hematoma at the puncture site was the most common immediate complication in both groups, classified as Type I, with incidence considered high, but all with spontaneous resolution, without intervention. A prospective cohort study using compressive dressing with gauze and elastic bandage positioned for four hours after diagnostic coronary angiography showed Type I hematoma in 7.5%, and Type II hematoma in 2.4% of the 120 patients evaluated with vascular ultrasonography after the removal of the dressing^(^ ^17^ ^)^.

The compressive dressing made with a sterile gauze cushion and adhesive tape was the device used in this study to obtain hemostasis. Its efficacy as an instrument of patent hemostasis is questionable, that is, it is not confirmed if the pressure applied during compression is enough to avoid bleeding and at the same time to maintain anterograde flow in the artery. A prospective study that included patients undergoing diagnostic cardiac catheterization who remained with compressive dressing similar to the one used in this study for two hours revealed that the absent flow before the removal of the compressive dressing was the only independent predictor of RAO at follow-up^(^ ^18^ ^)^.

A small portion of patients in our study presented patent hemostasis after the positioning of the compressive dressing. A study was conducted to compare three techniques for radial artery hemostasis after ECAT and PCI using customized compression dressing, or pneumatic wristband (PW) or rotational wristband (RW), regarding compression time, RAO and patient satisfaction. The study showed a higher occurrence of RAO after 24 hours of the procedure and a lower level of satisfaction in patients with compressive dressing (p < 0.05). The presence of anterograde flow in the radial artery during the hemostasis reaching process represents a strong and independent predictor of arterial patency after catheterization^(^ ^10^ ^)^.

A multicenter, prospective and non-randomized study evaluated the occurrence of bleeding and hematoma at the puncture site during compression and after removal of the device, as well as the occurrence of hematoma and RAO on the seventh day after the procedure, using compressive dressing in 416 patients or pneumatic hemostatic wristband in 112 patients. The authors showed a higher incidence of bleeding in the group of patients who received compressive dressing with gauze and adhesive tape (13.4%), when compared to no occurrence in patients submitted to compression of the artery with a pneumatic wristband (p < 0.001). However, a greater occurrence of hematoma was observed after seven days of the procedure in the group that used a compression wristband (p < 0.001), without significant influence on RAO (p = 0.20)^(^ ^11^ ^)^.

Authors reported that the radial artery compression time is directly related to the occurrence of RAO. The arbitrary use of recommended times for dedicated devices, which allow patent hemostasis, for customized dressings that hinder the maintenance of anterograde flow may be inadequate, leading to higher RAO. Researchers evaluated the effect of duration of hemostatic compression on the incidence of RAO after transradial coronary intervention and found that patients who maintained compression of the radial artery with a pneumatic wristband for six hours had higher rates of immediate (p = 0.025) and late (p = 0.035) RAO when compared to those who remained with compression for two hours^(^ ^12^ ^)^. A randomized study evidenced that compression duration was a strong predictor of RAO, supporting the hypothesis that, in order to minimize radial injury, the compression time should be reduced^(^ ^19^ ^)^.

As in our study, authors evaluated the occurrence of hemostasis and vascular complications according to two different times of compression of the radial artery in a randomized study with 568 patients submitted to coronary angiography and percutaneous coronary intervention, and to compression of the radial artery with rotational wristband for 20 or 60 minutes, both with patent hemostasis. The authors identified that patients who remained compressed for 20 minutes and required compression reinforcement showed higher rates of RAO (p < 0.01) and hematoma (p = 0.015). The need for a further application of pressure in the presence of bleeding in the group that remained for 20 minutes was the only independent predictor of RAO (p = 0.04)^(^ ^13^ ^)^.

Authors evaluated the influence of three volumes of insufflation of the wristband that corresponded to the intensity of the compression and time of dwelling on the occurrence of RAO and found that the lower the intensity and the compression time, the lower were the RAO rates^(^ ^14^ ^)^.

We decided in the present study to propose a reduction of radial artery compression time before the difficulty of obtaining patent hemostasis when using compression with a customized dressing made with a gauze cushion, considering the hypothesis that a reduction in hemostasis time could lead to lower RAO rates, without increasing the other vascular complications.

In a randomized study with 600 participants, the use of a chitosan-based cushion with hemostatic properties, kept compressed on the site of the introducer after PCI, showed better hemostatic efficacy with less compression time (127.6 ± 33.0 minutes *versus* 181.6± 32.2; p < 0.001), and a lower incidence of RAO after 24 hours (5.4% *versus* 11.7%, p < 0.05), and after 30 days (5.0% *versus* 10.0%; p < 0.05) compared to the use of pneumatic wristbands^(^ ^20^ ^)^. It should be noted that the two types of compression were maintained for at least one hour until the first site evaluation. The compression times of this study were higher than the times found in our study, and this may be justified by the fact that our patients were submitted only to diagnostic procedures that required lower doses of anticoagulants, more conservative antiplatelet therapy, and, in most cases the use of introducer sheaths with smaller caliber 5F, which favor the compatibility between the diameter of the introducer sheath and the radial artery, a factor considered important in the prevention of RAO.

Although there was no statistically significant difference between the compression times proposed in the study with respect to the evaluated outcomes, the shorter compression time of the artery could be recommended for clinical practice because it favors a shorter hospital stay of the patient, with a lower workload of the nursing team and reduction of hospital costs. The limitations in terms of vacancies for hospital admission and the high turnover of beds in hemodynamic services are a reality that most institutions face. For this reason, measures that reduce hospitalization time without compromising patient safety should be implemented^(^ ^21^ ^)^.

Although a small proportion of the patients presented patent hemostasis after the positioning of the compressive dressing and presented a rate of immediate RAO of 12.5%, regardless of the study group, the incidence rate of RAO (6.9%) in the evaluation after 30 days of the procedure was close to the rates presented in studies on the same theme that used different devices. In a meta-analysis with more than 31,000 patients, the incidence of RAO assessed within 24 hours after the procedure was 7.7% of the patients and 5.8% in the 30-day evaluation^(^ ^9^ ^)^. In our study, even without the use of specific hemostatic devices with higher costs than CCDs made with gauze cushions and adhesive tape, we can consider the late RAO rates found here compatible with other studies, especially in the group that remained with compression for less time.

The fact that the study was conducted in a single site, that only patients undergoing diagnostic procedures were allocated, and the difficulty of blinding since allocated patients and professionals were aware of the time established for the permanence of the compressive dressing at the site of the puncture are factors that represent limitations of this study.

## Conclusion

The different compression times, either 30 or 60 minutes, applied on the radial artery after transradial coronary angiography did not significantly influence the occurrence of hemostasis and immediate complications.
